# A Sustainable Microwave-Assisted Process for Chemical Recycling and the Reuse of Epoxy Resin Matrices

**DOI:** 10.3390/polym17070989

**Published:** 2025-04-05

**Authors:** Fabrizio Cafaro, Francesca Ferrari, Gloria Anna Carallo, Antonio Greco, Alfonso Maffezzoli

**Affiliations:** Department of Engineering for Innovation, University of Salento, Via per Monteroni, 73100 Lecce, Italy; francesca.ferrari@unisalento.it (F.F.); gloriaanna.carallo@unisalento.it (G.A.C.); alfonso.maffezzoli@unisalento.it (A.M.)

**Keywords:** microwave, chemical recycling, epoxy resin, composites, green chemistry, zero waste, reuse

## Abstract

This work presents an optimized and sustainable chemical recycling method for epoxy resin matrices, which uses microwave-assisted reactions to achieve the complete recovery of the matrix without generating waste byproducts. The proposed method employs a green chemistry approach, with hydrogen peroxide (H_2_O_2_) and tartaric acid (TA) as the eco-friendly reagents. Microwaves are used to activate the chemical reaction, ensuring localized heating, reduced energy consumption, and shorter processing times compared to conventional thermal methods. Unlike most existing recycling processes, which focus on fiber recovery, this study emphasizes the recovery and reuse of the matrix, transforming it into a valuable resource for producing new thermosetting materials. The recovered matrix was characterized using FTIR and H-NMR analyses, confirming the presence of reactive functional groups that enable its reintegration into new composite matrix formulations. The process has also demonstrated environmental benefits and economic advantages due to the absence of any waste and the reduced need for virgin raw materials. This method addresses a critical gap in composite material recycling, paving the way for a circular lifecycle and advancing the principles of sustainability in materials engineering.

## 1. Introduction

Epoxy resins have emerged as a cornerstone material in the production of fiber-reinforced composites due to their unique combination of mechanical strength, lightweight properties, corrosion resistance, and excellent adhesion. These attributes make epoxy-based composites indispensable in diverse applications, ranging from high-volume sectors like wind turbine blades [1,2] and shipbuilding, primarily using glass fibers, to high-performance domains such as aerospace, where carbon fibers dominate [3,4,5,6,7]. Furthermore, their versatility extends to coatings, adhesives, and electronics, generating significant amounts of epoxy resin waste across various industries.

The increasing demand for composites made from epoxy resins (glass fiber-reinforced polymers (GFRPs) and carbon fiber-reinforced polymers (CFRPs)) is projected to grow steadily due to their expanding applications [1,8,9,10,11,12]. However, this growth highlights a critical challenge—the lack of efficient strategies for recycling or reusing thermoset composite waste [13]. Unlike thermoplastics, thermosetting resins like epoxy cannot be remolded once cured, complicating their end-of-life management. Consequently, the decommissioning of composite materials, particularly wind turbine blades, has brought this issue into sharp focus [11,14,15,16,17,18].

Existing recycling processes for thermoset composites can be broadly categorized into mechanical, thermal, and chemical methods [12]. Mechanical recycling involves grinding the composite material into filler powders, which often suffer from a loss of fiber strength and aspect ratio, reducing their usability in high-performance applications [19]. Thermal recycling, including pyrolysis and fluidized bed reactors, primarily focuses on recovering fibers. However, these methods often require high energy inputs, generate toxic byproducts, and lead to the significant degradation of fiber properties [20]. Chemical recycling has gained attention as a more sustainable alternative, with various approaches utilizing supercritical fluids or acid-based reactions to recover fibers. However, these methods often involve harsh chemicals or high-temperature conditions, making them less environmentally friendly [19,21,22]. The current state-of-the-art way of recycling CFRPs is by pyrolysis. However, through the pyrolysis process, the polymer used in the CFRPs, cannot be recovered and reused. In most publications, the focus on CFRP recycling was on the recovery of the more valuable carbon fibers. The polymer matrix is mostly burnt off, in the case of pyrolysis, or disposed of [1]. Therefore, matrix materials, particularly epoxy resins, have been largely neglected, leaving significant potential untapped. Circular polymer solutions are possible through the increase in resource efficiency via the reduction in hazardous chemical use and utilizing more bio-based and recycled systems [23]. While some studies have explored resin recovery, the approaches are limited in scope or efficiency: a low percentage of the resin recovered in the new proposed material, the use of dangerous substances, and the production of wastes [6,24,25,26]. Notably, microwaves have been employed in recycling processes to achieve localized heating, improve energy efficiency, and reduce processing times. For instance, microwave-assisted pyrolysis has been shown to decrease waste volume by 73% [10], and fiber detachment has been achieved with minimal property loss [27]. However, the reported works only focus on carbon fiber recovery, which is mainly driven by economic issues, whereas comprehensive strategies that address matrix recovery, while adhering to principles of green chemistry, remain scarce. As indicated in different works [27,28], compared to chemical and thermal recycling, microwave-assisted chemical recycling (MACR) is environmentally friendly and cost-effective. LCA has indicated that the energy cost of MACR is 16 times and 30 times lower than that of chemical and pyrolysis, respectively. MACR reduces the environmental burdens associated with CFRP waste and virgin carbon fiber production. The MACR process has demonstrated improved recycling carbon credits, indicating energy savings and a decreased demand for VCF production [28]. Therefore, this study aims to recover the epoxy matrix through the optimization of a microwave-assisted chemical recycling process originally proposed by Zabihi for the recovery of CF [19]. As Thomas reports in his work [23], reducing plastic pollutants by extracting value added products from waste can help produce competitive sustainable polymers. The recovered resin (CR), formed by tartaric acid and depolymerized resin, is characterized and evaluated for its potential reuse in the production of new thermosetting matrices, paving the way to a more sustainable life cycle for composite materials. In fact, this work foresees its integral reuse for the production of new thermosetting matrices, mixed in various percentages with virgin epoxy resin.

## 2. Materials and Methods

### 2.1. Materials

L-Tartaric Acid (TA) and Hydrogen peroxide (35 wt% H_2_O_2_, 130 volumes) were provided by Sigma Aldrich (Darmstadt, Germany). A DiGlycidyl Ether of Bisphenol-Abased epoxy resin, Polar Bear, purchased by R*Concept (Barcelona, Spain), was used. This resin was chosen because it is 28% of plant origin and is liquid at room temperature; this is necessary to facilitate the mixing process with the curing agent [29]. Isophorone diamine (IPDA), a common hardener promoting curing at room and medium temperatures in several applications (Sigma Aldrich, Darmstadt, Germany), was used.

### 2.2. Methods

#### 2.2.1. Sample Production

The virgin epoxy resin (VIR) sample was produced by mixing epoxy monomer with IPDA (22 phr—stoichiometric ratio), then pouring it into a mold (35 × 10 × 2 mm), and finally curing was performed in a forced convection oven at 80 °C for 2 h followed by post-curing at 120 °C for 1 h. The chemical residue (CR), obtained from recycling the VIR sample, as described in the following section, was used for the production of new samples. This was carried out by manually mixing CR and VIR in different ratios (Table 1). Then, 22 phr of IPDA, calculated according to the amount of VIR used, was added to the mixture [29]. Curing was performed at 80 °C for 2 h followed by post-curing at 120 °C for 1 h. In order to compare the chemical recycling approach to mechanical recycling, samples were produced with the same composition as the previous ones, but the cured virgin resin was ground (GR) and mixed with uncured resin (Table 1). Afterward, samples made of liquid and solid resins were mixed with IPDA and cured following the same procedure used for the virgin resin.

#### 2.2.2. Optimization of Recycling Process

The solution used for the recycling process was prepared by mixing at room temperature for 30 min, 35 wt% hydrogen peroxide, and tartaric acid (a 3:1 weight ratio [19]), using a magnetic stirrer at 300 rpm. A resin sample of 0.81 g was immersed in 9 g of solution (corresponding to a 1:11 weight ratio), then the mixture was mixed gently and placed in the microwave oven (Sharp Corporation, Sakai, Osaka, Japan—max power 800 W). Recycling cycles were run in sequence, and in each cycle the solution completely evaporated, which required its replacement at the beginning of each cycle. In order to evaluate the weight loss that occurred, the resin sample was recovered at the end of the cycles, washed in distilled water, and dried for 24 h at 50 °C.

The sample weight loss (WL) was calculated by measuring the weight of the sample after the recycling test (RW), made up of three cycles, and comparing it with the initial weight of the virgin sample (IW):

WL % = (RW/IW) × 100 (1)

The microwave power was adjusted to minimize energy consumption and the number of process cycles while preventing the excessively rapid evaporation of hydrogen peroxide. Tests were conducted at power levels of 160, 240, 320, and 400 W. Lowering the microwave power delayed the onset of the hydrogen peroxide dissociation reaction, extending the process time. To accelerate the reaction, a food-grade yeast was introduced into the mixture as a natural catalyst. Yeast consists of an alkaline component (sodium bicarbonate), an acidic salt (tartaric acid salt), and an inert starch. The addition of yeast accelerates the dissociation of hydrogen peroxide into oxygen gas and water. The yeast makes it possible to shorten the reaction time, thus promoting the dissociation of the hydrogen peroxide which would otherwise only occur through the thermal activation (when approximately 80 °C is reached) caused by microwaves. The yeast (1.5 wt% solution) was therefore added to the recycling solution, mixing it gently before placing the mixture in the microwave oven. The progress of the reaction is evidenced by a rapid evolution of O_2_ and CO_2_ as bubbles.

Furthermore, a comparison between the microwave and conventional oven was also performed. Tests were performed at different temperatures (100–150 °C) using a vented oven (Carbolite, Thetford, UK—3000 W). The duration of each cycle at 100 °C is 30 min, at 150 °C it is 15 min, and at the end of each cycle the weight loss was calculated.

All the tests were performed on as-produced resin samples (35 × 10 × 2 mm) that had not previously been ground, since this would make it possible, when recycling the whole composite, to preserve the physical and geometrical characteristics of the fibers [30].

**Figure 1:**
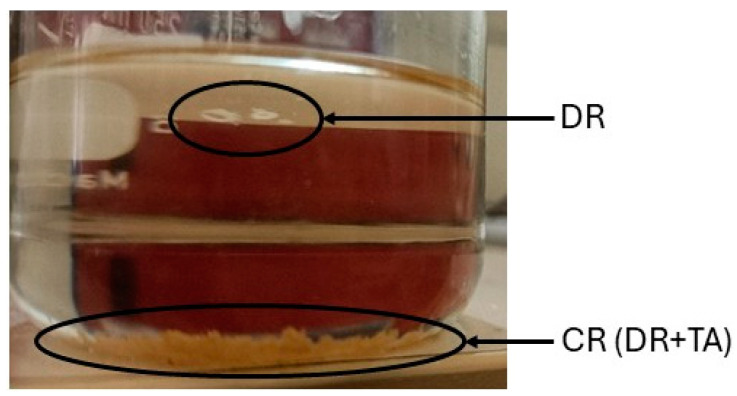
Separation process of CR in chloroform.

As a further step for improving the recycling process, the chemical residue (CR), which is made of resin and tartaric acid, was further separated in chloroform—the CR collected from the recycling process was immersed in chloroform for 24 h (Figure 1). The depolymerized resin (DR) that separated from the tartaric acid was collected and dried at 50 °C for 24 h and then analyzed.

#### 2.2.3. Testing Methods

A Jasco 6300 FT-IR spectrometer (JASCO Corporation, Tokyo, Japan) equipped with an ATR Pro One X with a ZnSe crystal was used for FT-IR analysis to characterize the samples and the recycling products (700 and 3500 cm^−1^; 48 scans; 4 cm^−1^ resolution).

H-NMR analysis was performed with a Bruker Avance III spectrometer (Bruker, Karlsruhe, Germany) operating at 400.13 MHz for a 1H observation and at a temperature of 300.0 K, equipped with a BBI 5 mm inverse detection probe incorporating a *z*-axis gradient coil. The sample was dissolved in deuterated methanol.

Mettler Toledo 822 (Greifensee, Switzerland) was used for DSC analysis—heating from 0 °C to 150–200 °C (10 °C/min), then a two-minute temperature hold, and cooling (10 °C/min) back to the starting temperature. A second heating scan, at the same heating/cooling speed, was used to evaluate the T_g_ of the cured samples.

A Lloyd LR5K dynamometer was used to measure the flexural properties of the samples (four replicates on each sample), according to ASTM D790-17 [31], via three-point bending tests.

## 3. Results and Discussion

### 3.1. Recycling Process

For each microwave power level, the holding time of the solution was limited by its complete evaporation, which was achieved after 11.5, 5.5, 3.3, and 2.6 min for microwave powers of 160, 240, 320, and 400 W, so that each cycle set in the microwave oven was equal to the time required for evaporation. In order to achieve the complete dissolution of the matrix, the number of required cycles, for any microwave power except 400 W, was equal to 10. When using a microwave power of 400 W, the very short holding time (i.e., 2.6 min) required an additional cycle to achieve complete sample dissolution. The effect of yeast addition was assessed for a microwave power of 320 W. As can be observed in Table 2, the yeast made it possible to save about 10% of the time needed for the complete dissolution of the matrix. The time needed for the complete dissolution of the matrix is reported for different microwave powers.

After each cycle, the solution was refilled to compensate for its evaporation—for even cycles (2, 4, 6, 8, and 10) only hydrogen peroxide was added. For odd cycles, the complete solution, including TA and yeast, was added. This was carried out in order to reduce the amount of TA residues in the degraded compounds.

Based on the data reported in Table 2, 320 W was chosen as the optimal power level, because it made it possible to reduce the process time required to obtain the complete degradation of the resin (with the same number of cycles) and therefore the consumption of hydrogen peroxide, TA and yeast.

**Figure 2:**
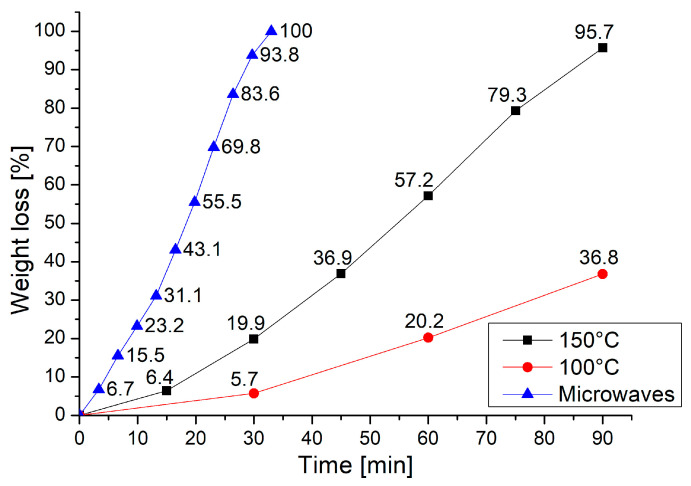
The weight loss trend of the resin during the test with conventional heating compared to the results obtained using microwave heating at 320 W.

A comparison between microwave and conventional heating was performed by repeating the same procedures in a vented oven—after 90 min at 100 °C, a weight loss of 36.8% was observed, while at 150 °C the almost complete degradation of the resin was achieved (weight loss = 95.7%). Figure 2 shows that, in 33 min, the time required to achieve complete dissolution of the resin by microwave at 320 W, only a 25% weight loss was achieved by heating in the oven at 150 °C. This clearly indicates that better performance is achieved when microwave heating is used.

### 3.2. FTIR and H-NMR Analysis

**Figure 3:**
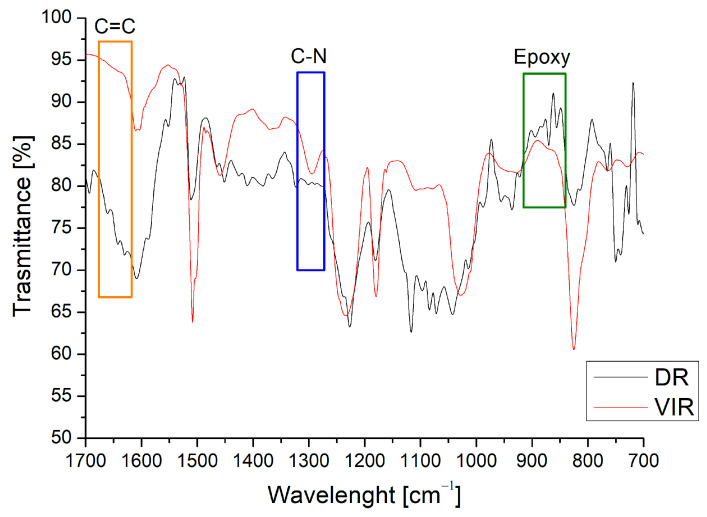
Comparison between FT-IR spectra of VIR and DR.

Figure 3 shows the spectrum of the DR, obtained after the separation of the TA from the recovered matrix, compared with that of the VIR sample. The peak at around 1300 cm^−1^ (the blue area in Figure 3), attributed to the C-N bond [32], is clearly evident in the VIR sample, but disappears in the DR sample. In addition, the epoxy peaks are clearly evident on the DR spectrum (the green area in Figure 3) in the range of 840–910 cm^−1^. Similarly, a barely visible peak is observed for DR around 1650 cm^−1^, relative to C=C. Both observations demonstrate that the chemical recycling mainly causes the cleaving of the C-N bonds, and the regeneration of epoxy and C=C bonds, making the DR material suitable for further curing in the presence of amine or by radical chain polymerization.

However, the developed approach for TA/DR separation is at the moment not in line with green chemistry principles, since it requires solvents and is very time-consuming. In addition, the presence of TA in the chemical residue is believed to potentially contribute to and accelerate the curing reaction of the epoxies [33]. Therefore, for the following thermal and mechanical characterization, the VIR epoxy was mixed with CR, which is made of depolymerized resin and TA. The chemical residue was collected, dried (for 24 h at 50 °C), and analyzed by FTIR and H-NMR. No waste products were eliminated.

**Figure 4:**
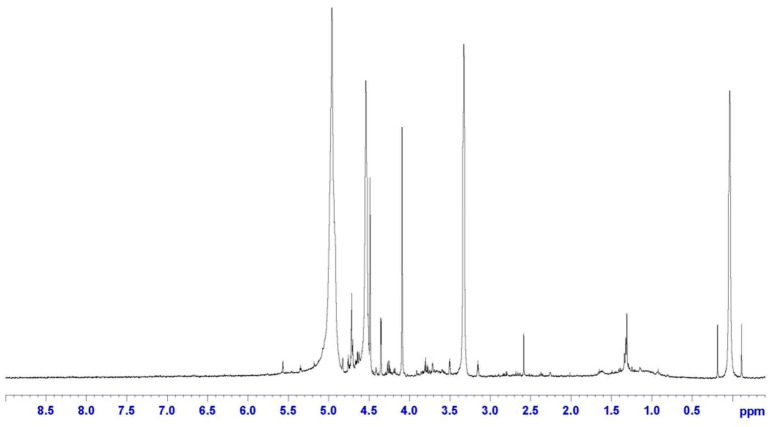
H-NMR spectrum of CR.

The presence of epoxy rings on CR was confirmed by the H-NMR analysis reported in Figure 4, showing the typical signal at δ = 2.6 ppm. Also, the chemical shift related to the methylene groups adjacent to these epoxies is evident at δ = 1.4 ppm. Finally, double bonds (C=C) are observed at δ = 5 ppm, which confirms the results from the FTIR analysis, highlighting the further reactivity of CR.

The FTIR spectra of the cured samples, consisting of 10% CR and virgin resin (VIR_CR_1), are compared to the spectrum of the chemical residue (Figures S1–S3). The main differences between the two samples are observed in the 920 and 840 cm^−1^ range; the peaks in the CR spectra, attributed to the epoxy rings, disappear in the VIR_CR_1 spectra, indicating that CR participates in the reaction. In order to quantitatively evaluate the epoxy groups present in the CR, we performed titration tests according to Method A ASTM D1652-97 [34], comparing the results with those obtained for virgin epoxy resin; the epoxy equivalent (EE) for CR is three times lower than that of the virgin resin.

On the other hand, the peaks relative to N-H bending at 1600 cm^−1^, N-O stretching at 1500 cm^−1^, and C-N at 1300 cm^−1^ are only visible on VIR_CR_1. Relevantly, the C-N bond is formed as a consequence of the epoxy ring reaction, as already discussed for Figure 3.

Finally, the spectra of VIR_GR_1 and VIR (Figures S2 and S3) overlap almost perfectly, indicating that, unlike CR, GR acts as an inert filler during epoxy curing.

### 3.3. Thermal Characterization

Initially, due to the presence of epoxy rings, the as-separated CR was used for the production of solid samples after curing with IPDA. However, the DSC analysis performed on the cured CR highlighted a very low T_g_ value (around 32.6 °C, as reported in Table 3), which makes the material unsuitable for use as a composite matrix. The T_g_ of the sample DR, after curing in the presence of amine, is around 82.5 °C. The difference in T_g_ between the two materials demonstrates that the tartaric acid present inside the CR negatively affects the thermal properties of the cured systems, which requires the mixing of the CR with the virgin resin in order to obtain a material with a higher T_g_.

All cured CR/VIR samples show a decrease in the glass transition temperature in comparison to VIR. In addition, T_g_ decreases with increasing CR content (Table 3). However, the glass transition temperature measured for the composition with 40% of CR is still high enough (about 82 °C) for its further use as a composite matrix in non-critical structures such as in automotive and aircraft interior applications [35]. The results of Table 3 are in line with those reported by Cho et al. [1], who also observed a T_g_ reduction of about 35 °C for samples of DGEBA mixed with 10 wt% of a matrix recycled by solvolysis. The T_g_ decrease in the sample containing CR can be explained by considering the reduced EE of the CR compared to virgin resin. The resulting lower degree of the crosslinking of the samples containing CR has the direct effect of increasing molecular mobility and, therefore, reducing the T_g_.

The glass transition temperatures of the samples containing GR increase as the GR content increases. The increase of T_g_ with the increasing amount of GR can be explained by considering that the inert filler GR reduces segmental mobility, thus increasing the T_g_.

### 3.4. Mechanical Characterization

**Figure 5:**
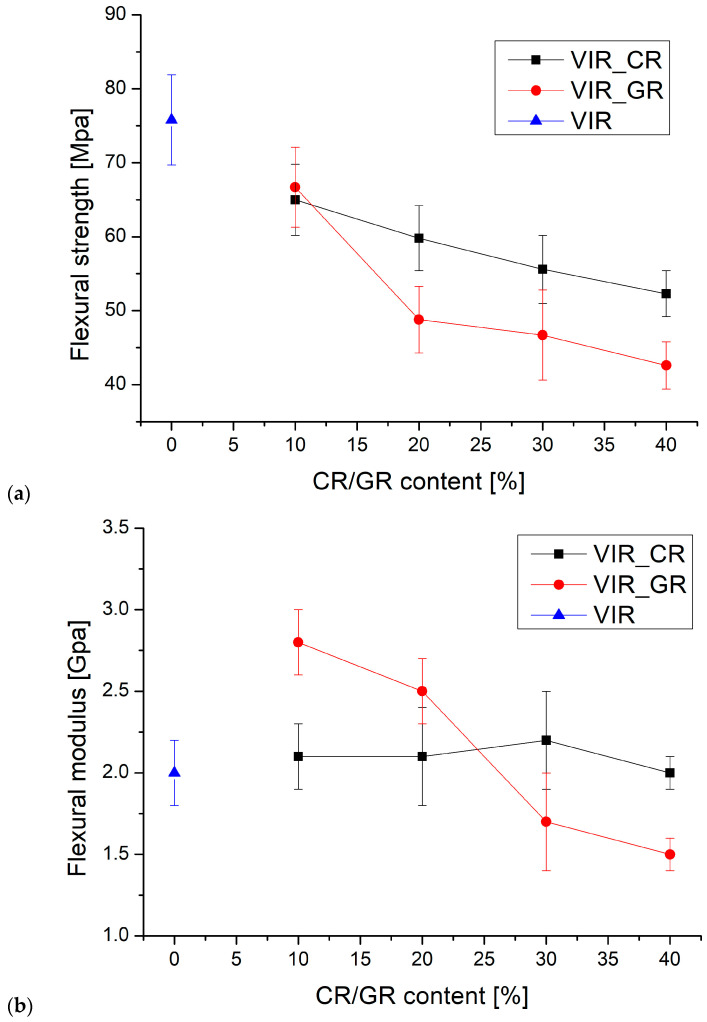

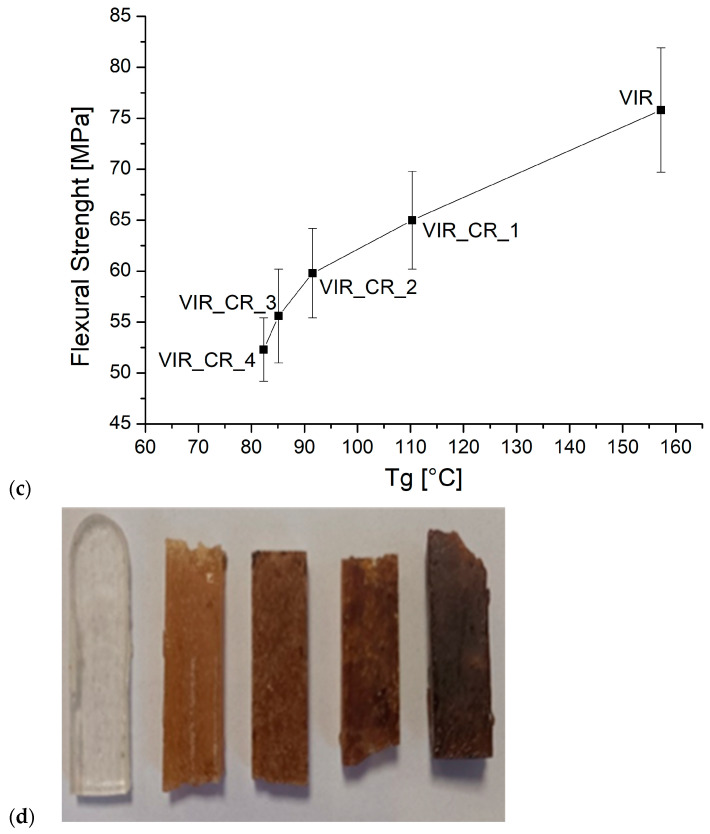
(**a**,**b**) The trend of flexural strength and flexural modulus versus CR and GR contents, (**c**) the correlation between flexural strength and T_g_, and (**d**) the samples after the flexural test (to the left: VIR, VIR_CR_1, VIR_CR_2, T VIR_CR_3, and VIR_CR_4).

Finally, the cured samples were tested by three-point bending test. Figure 5 shows the average values of flexural strength (a) and modulus (b) versus the CR and GR contents.

Also, in view of the error bars associated with the measurements, two-way analysis of variance (ANOVA) was performed by considering the type of recyclate (CR or GR) and the amount of recyclate as the sources of the variations. The significance of each factor was tested by calculating the F value, as the ratio of the variance between the means to the variance of the experimental error. The F value was then used in order to calculate the corresponding *p* value, which was then compared with the confidence level, α = 0.05. According to ANOVA, *p* > α corresponds to the null hypothesis (the equivalence of the means), whereas *p* < α indicates that the population means are significantly different [36].

For the flexural strength, the results of the ANOVA, summarized in Table 4a, highlight that, for both Factor 1 and Factor 2, *p* < α indicates that the effect of the recyclate type and amount on the flexural strength is statistically significant. In particular, the results reported in Figure 5a show that the flexural strength of the samples containing CR is higher than that of the samples containing GR, and that for both types of recyclates the flexural strength decreases as the amount of recyclate increases. Also, the *p* value calculated for the interaction between factors is lower than α, indicating that the amount of recyclate has a specific effect for different recyclate types. It is equally evident that the effect of the amount of recyclate is more significant for GR than for CR.

For the flexural modulus, the results of the ANOVA, summarized in Table 4b, highlight that for Factor 1, *p* > α indicates that the effect of recyclate type on the flexural modulus is not statistically significant. In contrast, for Factor 2, *p* < α indicates that the amount of recyclate has a statistically significant effect on the flexural modulus. Also, the *p* value calculated for the interaction between factors is lower than α, indicating that the amount of recyclate has a specific effect for different recyclate types. In particular, the results reported in Figure 5b coupled with the ANOVA indicate that for CR the modulus is substantially independent of the amount of recyclate, whereas for GR, the modulus significantly decreases with the amount of recyclate.

Therefore, it is possible to conclude that the flexural modulus is barely influenced by the presence of the recycled matrix, as also found by Cho for a CFRP matrix recovered by solvolysis [1]. However, comparing the results of the flexural strength test with those reported in [1], it is clear that much better performance is achieved by the present approach. In fact, Cho observed a strength reduction of about 80% with the addition of 5% of a recycled matrix to the virgin matrix, which is much higher than for any of the formulations reported in Figure 5a, despite the much higher recyclate content.

Very interestingly, as highlighted by Figure 5c, a strong correlation exists between the flexural strength and T_g_, which is in turn strictly dependent on the degree of the crosslinking of the polymer. Therefore, the embrittlement observed due to the addition of CR, can be mainly attributed to a reduction in the degree of crosslinking as the amount of CR increases.

## 4. Conclusions

This work assessed an innovative method for the recovery of epoxy matrix from GF or CF composites. The method, previously developed for the recovery of CF, was optimized in order to maximize the yield and minimize the recycling time and energy consumption in view of the complete reuse of the epoxy matrix. The best recycling conditions for microwave-assisted chemical recycling were identified. The chemical residue from the recycling process is characterized by the presence of epoxy and C=C reactive groups, which enables it to react in the presence of an amine hardener. However, the very low T_g_ found for cured CR requires mixing with different amounts of virgin resin in order to improve its thermal properties. The addition of CR involved a reduction in glass transition and flexural strength compared to virgin resin, which suggests its potential use for the production of fiber-reinforced composites for the automotive sector or for non-structural components. It is worth mentioning that the strength reduction obtained in this work is much lower than that reported for an epoxy matrix recovered by solvolysis. In addition, the proposed approach proved to be more efficient than conventional mechanical recycling in view of its potential use in blends with virgin resin. Some preliminary tests also showed that an improvement of thermal properties, and therefore a potential improvement of mechanical properties, can be achieved by further separating the chemical residue from tartaric acid to obtain the depolymerized epoxy. The next work will therefore be focused on the optimization of the last separation step, which is believed to potentially lead to the closed-loop recycling of epoxies for high-performance applications.

## Figures and Tables

**Table 1:** Composition of samples containing the chemical residue (CR) or the ground virgin epoxy resin (GR).

Samples	VIR [wt%]	CR/GR [wt%]	IPDA [phr on VIR]
VIR_CR_1	90	10	22
VIR_CR_2	80	20	22
VIR_CR_3	70	30	22
VIR_CR_4	60	40	22
VIR_GR_1	90	10	22
VIR_GR_2	80	20	22
VIR_GR_3	70	30	22
VIR_GR_4	60	40	22

**Table 2:** Total process time (with yeast) and number of cycles as a function of microwave power.

Microwave Power [W]	Total Process Time [min]	Number of Cycles
160	115	10
240	55.5	10
320	36.2 (no yeast)	10
320	33	10
400	28.5	11

**Table 3:** T_g_ values found with DSC analysis.

Samples	T_g_ [°C]
CR	32.6
VIR	127.2
DR	82.5
VIR_CR_1	110.3
VIR_CR_2	91.5
VIR_CR_3	85.1
VIR_CR_4	82.3
VIR_GR_1	132.6
VIR_GR_2	139.6
VIR_GR_3	141.7
VIR_GR_4	142.5

**Table 4:** (**a**) Two-way ANOVA analysis results for flexural strength; (**b**) two-way ANOVA analysis results for flexural modulus.

**(a)**	**Degrees of Freedom**	**Mean Square**	***p***
Factor 1 (type of recyclate)	1	389	2.74 × 10^−4^
Factor 2 (amount of recyclate)	4	1370	2.74 × 10^−16^
Interaction	4	88.4	1.31 × 10^−2^
Error	40	24.2	
**(b)**	**Degrees of Freedom**	**Mean Square**	***p***
Factor 1 (type of recyclate)	1	5 × 10^−3^	0.75
Factor 2 (amount of recyclate)	4	7.9 × 10^−1^	4.84 × 10^−8^
Interaction	4	7.2 × 10^−1^	1.57 × 10^−7^
Error	40	4.8 × 10^−2^	

## Data Availability

Data are available on request.

## Supplementary Materials

The following supporting information can be downloaded at: https://www.mdpi.com/article/10.3390/polym17070989/s1, Figure S1: FTIR of CR—VIR_CR_1; Figure S2: FTIR of VIR—VIR_GR_1; Figure S3: FTIR of CR—VIR_CR_1—VIR—VIR_GR_1.
