# Supplementary figures and images for: A Sustainable Microwave-Assisted Process for Chemical Recycling and the Reuse of Epoxy Resin Matrices
Source: Polymers (Basel). 2025 Apr 5;17(7):989. doi: 10.3390/polym17070989 (PMC11991300; doi:10.3390/polym17070989)

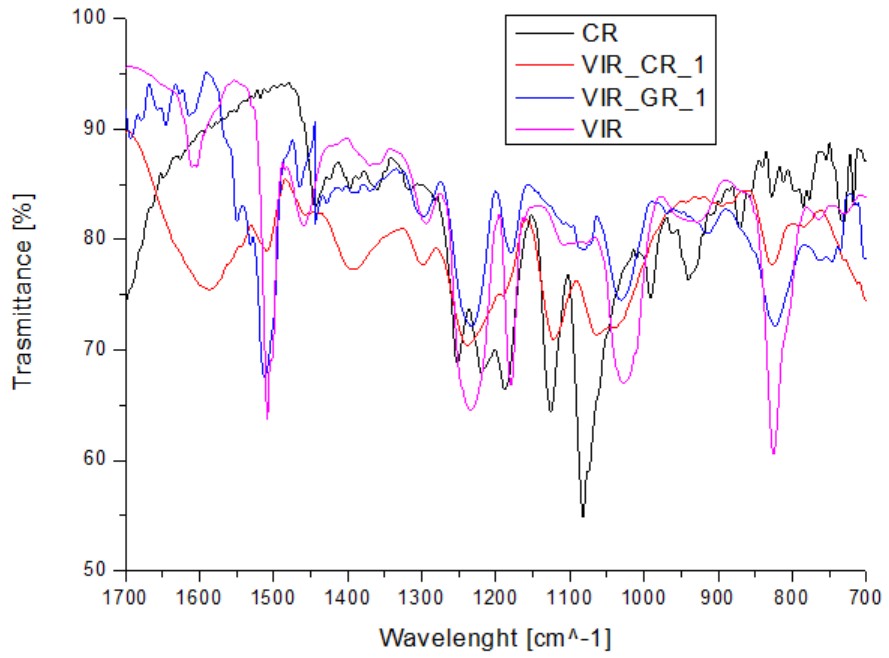

Supplement: Supplementary file 1 [file polymers-17-00989-s001.zip › Figure S3 - FTIR of CR-VIR_CR_1-VIR-VIR_GR_1.jpg]

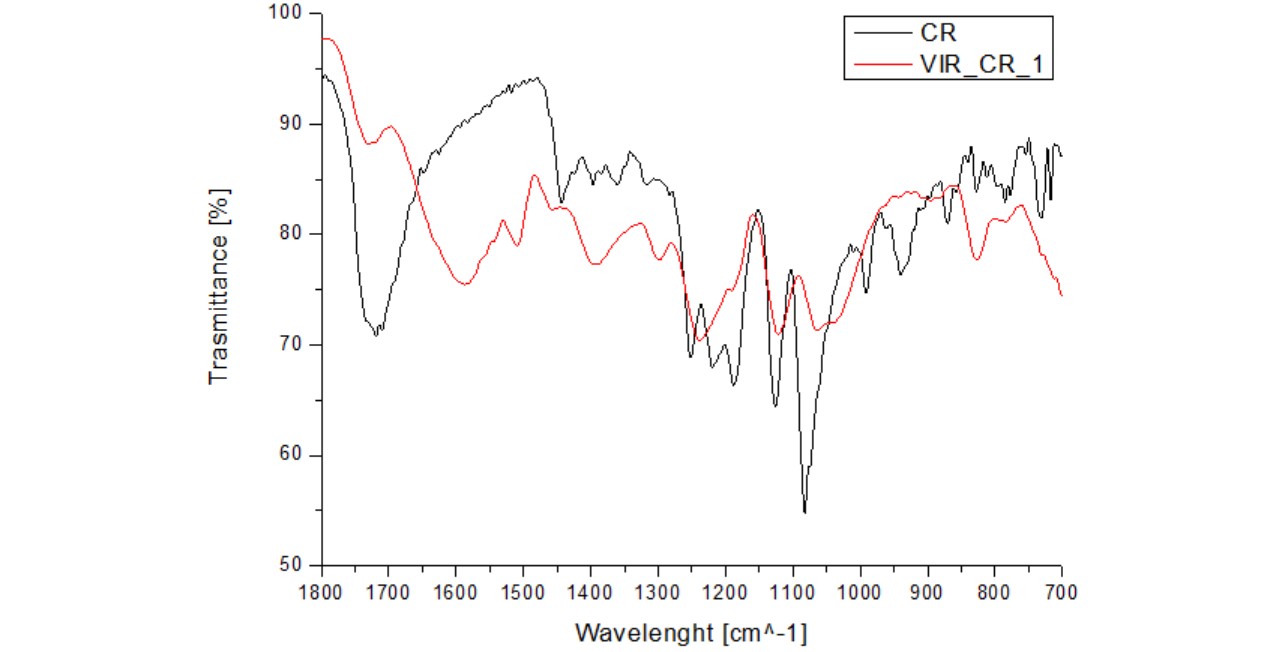

Supplement: Supplementary file 1 [file polymers-17-00989-s001.zip › Figure S1 - FTIR of CR-VIR_CR_1.jpg]

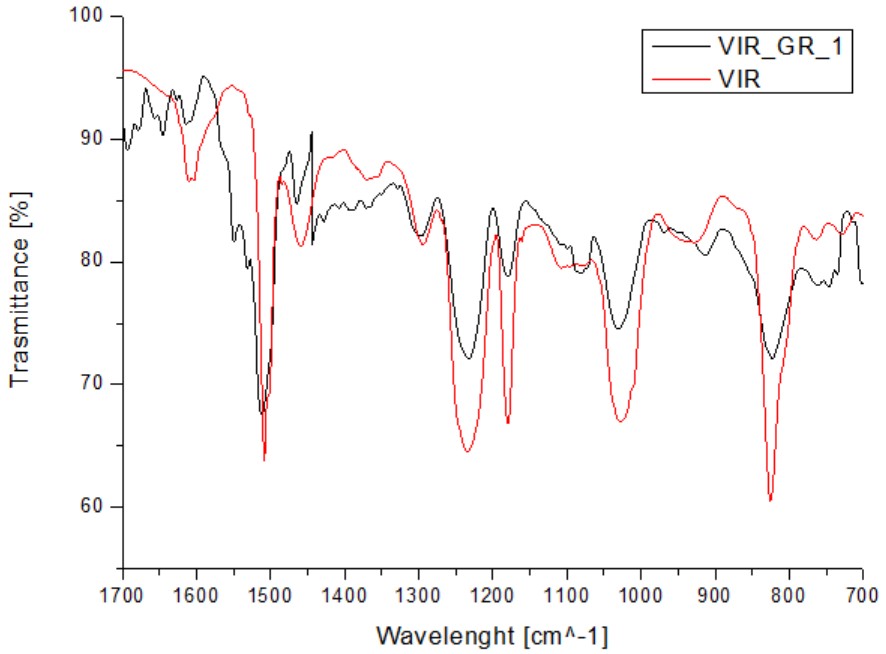

Supplement: Supplementary file 1 [file polymers-17-00989-s001.zip › Figure S2 - FTIR of VIR-VIR_GR_1.jpg]
